# Items

**Published:** 1882-05

**Authors:** 


### Items.

PROF. HAMMOND says that "if a drop of solution of nitro-glycerine in alcohol, in the proportion of one part in a hundred, be placed on the tip of the tongue, a sensation of fullness and pain in the head (mainly in the frontal region) is experienced in the course of three or four minutes." A few moments later "the head feels as if it is about to burst open." We are not surprised at this last statement. It is in keeping with the reputation nitroglycerine has achieved—though not so much premonition is usually given. For our own part we do not propose to have anything to do with this remedy. It may be prompt, but is not reliable. A patient with a pill of it in him would be but a moving volcano—quiet, but with a liability to eruptions of a serious character. Nor would we, were we a druggist, make a pill, powder, or solution of it. As "Uncle Remus" would say, it is altogether too "tetchy;" and when it *goes off* it is apt to carry too much with it.

---

THE following extract is taken from the Southern Practitioner, in a review of the Transactions of our State Society: "The handsomest volume of society transactions we have examined has been forwarded us with the compliments of the efficient and accomplished Secretary Dr. A. Sibley Campbell. The Georgia Association is as live and full of progress as the most exacting could require. In the beautifully printed volume before us, executed in a style that will attract attention and excite admiration, even when compared with the best efforts of the most successful publishing houses on either side of the Atlantic, will be found a full report of the minutes and papers of the 32d Session held at Thomasville, Ga., April 20 *et seq*., 1881."

---

THE *Michigan Med. News* says: A coroner's jury verdict lately read: "The deceased came to his death by excessive drinking, producing apoplexy in the minds of the jury." In this part of the vineyard, there seems to be always something ready to produce an obtunding apoplexy in the minds of a coroner's jury. Such a jury would as soon condemn a suspected man to death, as to say that the victim died from natural or unknown causes, one or the other. There may have been witnesses to the death, or there may not; but don't deprive the coroner of his fee! Further, he may have a medical friend who needs a fee. Let that friend thrust his finger into a bullet hole, and call it a *post mortem* examination. Who cares? The law is satisfied, the thing looks official, and all who receive fees are happy.

---

THE *Boston Journal of Chemistry* says that salicylic acid, though forbidden for this purpose in France, is used in Germany and other countries quite freely for the preservation of "alimentary substances, as it does not in any way affect either the good quality of the articles preserved or the health of the consumer." One drachm of chrystalized acid to every three pounds of fruit is recommended. "Raw fruit can be preserved a long time when wrapped in clean salicylated paper. The juice of berries or any other kind of fruit will keep well for more than a year if treated immediately with salicylic acid at the rate of one drachm to three pounds of juice. This leaves the addition of sugar optional."

---

WE all respect the crusade Mr. Bergh, of New York, has made for the prevention of cruelty to animals. We do not object if all the horses in New York should, as has been said they do, raise their hats whenever they meet him. We are ready to apologize for him if he goes to some extremes upon this subject. We can be patient with him when he proposes that all oysters on the half-shell shall be chloroformed before being swallowed. But when he undertakes to inaugurate or lead an anti-vaccination craze, then we say it is time that he be required to answer to a charge of lunacy.

---

THE Medical Times: It was therefore with much satisfaction that we heard Dr. D. Hayes Agnew, in clear, forcible language, upon the stand, affirm that he was no expert in insanity, and that his testimony should not be considered as coming from such a one. Modesty like this adorns him who has it, aids in the obtaining of justice, and exalts the profession.

We endorse this sentiment. Most physicians should be honest enough to make a similar confession. They would thereby tell the truth, and avoid humiliation at the hands of cheap lawyers; and the cause of justice would probably be promoted by such declaration.

---

THE article of Dr. Herrick, in the Michigan *Medical News,* brought forth a generous sprinkling of criticism. He boldly argued that it often is the duty of the physician to recommend the prevention of conception, and the means therefor, in certain instances of married life. His views, or rather his boldness in expressing them, are novel; but he writes clearly, to the point, and with no little common sense. He shrewdly says that the proper prevention of conception, not always immoral, often prevents intentional abortion which must be always criminal.

Another medical society has been organized in this city and named The Gate City Medical Society. It is composed of the younger men of the profession. Some one irreverently dubbed it the juvenile society. No city of Atlanta's size can claim a larger number of able and earnest young physicians. Their object and endeavor in this step is worthy of commendation. Some time since we stated that our city had fallen into a state of lethargy in regard to societies, andpredicted a reaction. As two have been organized within a few weeks, we may set up a claim to prophecy

---

The Western Medical Reporter, in speaking of a new medical colldge building, says: "The basement will contain in addition to a commodious suite of janitor's apartments, full dining and kitchen facilities for furnishing day-board to an almost unlimited number of students, one unusual feature of this floor being a capacious vault refrigerator for the reception of as many as a hundred dead bodies for dissecting purposes." It seems to us that dinner and dead bodies are rather badly mixed in this sentence. The last three words, however, may somewhat relieve apprehension.

---

At the meeting of the Southern Illinois Medical Aesociation, Dr. L. Dyer in an address on the Reminiscences of Professional Life, said: "He remembered well when qui nine was first introduced in his part of the country. He sent twenty miles to purchase a drachm, for which he paid a dollar, and prescribed it in one grain doses, which was supposed to be a medium dose. He never knew whether it did any good or harm." Dr. L. D. Ford, of Augusta, Ga., could wiite a volume on the early introduction of quinine.

---

Gaillard's Journal says the fee of fifteen thousand dollars each, which it is proposed to pay to Drs. Hamilton and Agnew for their services in President Garfield's case, is niggardly. We are disposed to agree with Dr. Gaillard. It would take more than that to act as a kind of soothing syrup for the privilege of playing the part of a target for a whole nation's "cussing." But the somewhat notorious

Dr. Bliss answers very well as a butt for some of this abuse.

---

Dr. J. J. Woodward, of the army, and President of the American Medical Association, has gone to Italy, and thence to Switzerland, for failing health. He has a leave of absence for eight months. He leaves unfinished his official work on the third (and last) volume of the "Medical History of the War of the Rebellion" (a bad name for a good work). He, therefore, will not preside at the coming meeting of the Association.

---

Caconemia is the name proposed by Prof. John H. Logan, of this city, for a disease hitherto designated by a variety of inelegant and inexpressive epithets. Caconemia, then, is a disease of grave importance in a pathological point of view, and from its insidious nature, as well as from the facility with which it seems to be propagated by ordinary articles of food, possesses special interest for sanitarians. We commend the communication of Dr. Logan, on this interesting theme, to our readers.

---

A lady complaining to her attending physician, during an attack of severe illness, that the taste of an important prescription, which she was taking, was disagreeable, received, in reply, the rather emphatic declaration, "Madam, we are not confectioners." This explanation was, apparently, satisfactory, as no further dissatisfaction with the flavor of the drug was expressed.

---

Dr. D. W. Yandell's pamphlet on Epidemic Convulsions treats of the "jerks" that attended the religious excitement that broke forth at the revivals of the pioneers of some of the Western States. The novelist, (and preacher) Edward Eggleston, gives vivid descriptions of them in some of his works.

---

"The Medical Press and Circular says: "An antivivisectionist who subscribes £20 yearly towards carrying on the antiscientific agitation, has been fined for maltreating an unoffending animal in his possession.—*Boston Med. and Surgical Journal.*

Simple Continued Fever.—Dr. Fothergill, in speaking of the following formula, says it will probably constitute, *par excellence*, the fever mixture of the future: R.—Acid hydrobrom., ʒi.; Syr. simplicis, ʒij.; Aq. ad., ℥i. M. Sig. One teaspoonful every hour. It is specially indicated where there is cerebral disturbance.

---

The *Boston Journal of Chemistry* asks, What are fallacies? We are disposed to amend by asking, What is it that is not a fallacy, when we read of the premature new remedies suggested, and of the multitudinous fancy preparations constantly thrust under the professional nose?

---

We direct attention to the number of pages of reading matter presented in each issue of The Register. Not a few journals with as large, or even larger subscription price, furnish not more than one-half or two-thirds as much; but endeavor to cover the deficiency by a display of thick paper.

---

As the secular press has extensively announced it, it is almost too late to mention the resignation of Professor S. D. Gross. He is seventy-seven years of age. The chair thus vacated will be divided between Drs. J. H. Brinton and S. W. Gross.

---

Salzer has been counting the fibres of the optic nerve. He counts four hundred and thirty-eight thousand. The number of cones in the retina he found to be three millions five hundred thousand.—*Ohio Med. Journal.*

That is just precisely the number we had guessed.

---

An Eclectic medical journal rakes certain regular physicians (professors) for attaching their names to certificates in the interest of some semi-quackish medicines. Surely, they will now amend their ways. Truly a deserved rebuke; but from such a quarter!

---

Syphilis of the Finger—In the *Independent Practitioner* for March, 1882, Dr. F. N. Otis reports eight cases of syphilis occurring in physicians, originating in infection of the finger in vaginal examinations.

Dr. Davis' article in our last issue, is worthy of attention from authors of treatises on practice of medicine. "Milk sickness" is a disease of interest to a large scope of country, and the literature of the subjeet is meagre.

---

The first woman graduate of medicine, so far as we can learn, that Georgia has ever produced, received her diploma recently from the Eclectic school, of this city. She was a young woman recently arrived from Germany.

---

The *Boston Medical Journal* contains a long article by Walter Channing, M.D., claiming that Guiteau is really insane, and advocating his confinement in an asylum for lunatics.

---

Dr D. Bethune Duffield, in an address at the commencement of the Detroit Medical College, "dropped into poetry" as easily as did the renowned Silas Wegg. It took eight pages to deliver him.

---

There were two hundred and forty-seven graduates at the recent commencement of the Jefferson Medical College. There were 630 students in attendance.

---

Seven hundred and nine doctors were let loose in Philadelphia at the recent commencements, against seven hundred and thirty-one the year before.

---

Dr. Martin (Med. Times and Gazette), uses fresh parsley leaves for drying up the flow of milk. They should be renewed several times a day.—*Detroit Clinic.*

---

There were more than six hundred applicants for the position of surgeon to the Western Railway, England. The salary is nearly three thousand dollars.

---

Dr. W. O. Roberts has been elected to the chair of Operative Surgery and Surgical Pathology in the Medical Department of the University of Louisville.

---

It is said the father of Oscar Wilde was an eminent aurist. This may account for the development of Oscar's ears.

